# Imported leishmaniasis in travelers: a 7-year retrospective from a Parisian hospital in France

**DOI:** 10.1186/s12879-021-06631-5

**Published:** 2021-09-15

**Authors:** Nesrine Aissaoui, Samia Hamane, Maud Gits-Muselli, Antoine Petit, Mazouz Benderdouche, Blandine Denis, Alexandre Alanio, Sarah Dellière, Martine Bagot, Stéphane Bretagne

**Affiliations:** 1 Laboratoire de Parasitologie et de Mycologie, Hôpital Saint-Louis, Assistance Publique-Hôpitaux de Paris (AP-HP), 1 Avenue Claude Vellefaux, 75475 Paris, France; 2 Université de Paris, Paris, France; 3 Service de Dermatologie, Hôpital Saint-Louis, Assistance Publique-Hôpitaux de Paris (AP-HP), Paris, France; 4 Département de Maladies Infectieuses, Hôpital Saint-Louis, Assistance Publique-Hôpitaux de Paris (AP-HP), Paris, France; 5 INSERM U976, Paris, France

**Keywords:** Cutaneous leishmaniasis, Visceral leishmaniasis, Quantitative PCR, Cytochrome *b* sequencing, *Leishmania major*, *Leishmania tropica*, *Leishmania infantum*

## Abstract

**Background:**

Leishmaniases are regularly seen in non-endemic areas due to the increase of international travels. They include cutaneous leishmaniases (CL) and mucocutaneous (MC) caused by different *Leishmania* species, and visceral leishmaniases (VL) which present with non-specific symptoms.

**Methods:**

We reviewed all consecutive leishmaniasis cases seen between September 2012 and May 2020. The diagnostic strategy included microscopy after May-Grünwald-Giemsa staining, a diagnostic quantitative PCR (qPCR) assay, and species identification based on sequencing of the cytochrome *b* gene.

**Results:**

Eighty-nine patients had a definitive leishmaniasis diagnosis. Nine patients had VL with *Leishmania infantum*. Eighty patients had CL. Twelve patients acquired CL after trips in Latin America (7 *Leishmania guyanensis*, 2 *Leishmania braziliensis*, 2 *Leishmania mexicana*, and 1 *Leishmania panamensis*). Species could be identified in 63 of the 68 CLs mainly after travel in North Africa (59%) with *Leishmania major* (65%), *Leishmania tropica/killicki* (24%), and *L. infantum* (11%), or in West Sub-Saharan Africa (32%), all due to *L. major*. The median day between appearance of the lesions and diagnosis was 90 [range 60–127].

**Conclusions:**

Our diagnostic strategy allows both positive diagnoses and species identifications. Travelers in West Sub-Saharan Africa and North Africa should be better aware of the risk of contracting leishmananiasis.

## Highlights

- Imported leishmaniases are regularly seen in non-endemic areas.
- Cutaneous forms are due to different species that need to be correctly identified for adapting treatment and epidemiologic purposes.
- The index of suspicion for the visceral form is often low because of the non-specificity of the clinical symptoms and the notion of travel in endemic areas often remote.
- The strategy, based on diagnostic quantitative PCR followed by sequencing for species identification, allows for rapid and safe diagnoses in a routine laboratory.

## Introduction

Leishmaniases are zoonotic and anthroponotic diseases caused by several protozoan species in the genus *Leishmania* that are transmitted by the bites of phlebotomine sand flies. They represent a major public health problem in endemic countries, with regular increases reported in the past decade [1–3]. Different species present with diverse clinical symptoms, with a certain degree of specificity in the clinical presentation depending on the species [4]. Leishmaniases are thus classified into cutaneous (CL), mucocutaneous (MC), and visceral leishmaniases (VL) [5, 6].

Whereas VL in non-endemic areas is usually seen in immunocompromised adult patients living or having lived in endemic areas, imported CLs are easily linked to international travels. In recent years, these imported CLs have been on the rise due to international tourism, military operations, and the influx of immigrants from endemic countries [7–9]. The French National Reference Centre reported a stable number of cases until 2012 [10], but now reports an annual increase in CL declarations, from 130 in 2013–2017 to 214 in 2018, mostly (90%) from North Africa (https://cnr-leish.edu.umontpellier.fr/files/2019/05/Rapport_CNRLeishmanioses_Act-2018.pdf). We were therefore interested in analyzing the data collected in our hospital located in northeast Paris.

We were also interested in validating our two-step diagnostic strategy, which includes a real-time quantitative PCR (qPCR) assay for the positive diagnosis targeting the consensus sequence of the highly repeated kinetoplast DNA, followed by the amplification and sequencing of a cytochrome *b* (*cytb*) gene fragment for species identification [11]. Indeed, the species identification step is necessary for making the best therapeutic decisions according to species [5, 6]. If VL is mainly due to *Leishmania infantum*, different species can be responsible for CL in both Latin America and the Mediterranean basin, and they have different progressions and treatment options [3, 12].

## Patients and methods

### Patients and sample processing

All patients with a positive leishmaniasis diagnosis seen in our laboratory at Saint-Louis Hospital in Paris, France between 1 September 2012 and 31 May 2020 were included. Clinical and epidemiological data, including the age, gender, country visited, length of stay, date of return, date of lesion(s) onset, clinical aspect, and anatomical site of the lesions, were collected, as were the treatments.

Skin scrapings or biopsies were performed by clinical microbiologists skilled in the diagnosis of CL. Thin smears were examined under a microscope after May-Grünwald-Giemsa staining. For VL, DNA was extracted from all specimens requested by the clinicians (i.e., bone marrow, blood, and/or tissue biopsies) using the QiA Symphony extraction kit (QIAGEN, Germany) following the manufacturer protocol. The molecular investigations have been previously described [11]. Briefly, a *Leishmania* diagnosis was confirmed by qPCR amplification of a 152-bp fragment of 18S rRNA. The quantitative results are expressed in the quantification cycle (Cq), with DiaControlDNA CY (Diagenode Diagnostics, Liège, Belgium) used as an internal control (IC). The presence of PCR inhibitors is excluded when the difference between the IC expected Cq value and the clinical sample IC Cq value is < 3. Additionally, the quantity of sampled human cells is checked by amplification and comparison with the single-copy human albumin gene. As previously proposed for the diagnosis of pneumocystosis [13], the number of human cells was considered sufficient if there were ≥ 1000 copies/mL of albumin DNA.

### Species identification

Species identification was performed by sequencing an 872 bp fragment of the *cytb* gene using two sets of primers as previously described [11]. The sequences were aligned and analyzed using the Geneious multiple sequence-alignment program and compared with reference sequences in the GenBank database using the BLAST algorithm. Single nucleotide polymorphisms (SNPs) were identified and numbered according to previously described SNPs [11].

### Statistical analyses

Statistical analyses were performed using SPSS 24 software (SPSS Inc, Armonk, NY). The Pearson chi-square test was used to compare variables with a confidence of 0.95.

## Results

### Leishmaniasis diagnosis and species identification

Overall, 292 and 662 patients were tested for a suspicion of CL and VL, respectively, during the study period. We obtained 89 *Leishmania*-positive samples, 80/292 (27.4%) for CL and 9/662 (1.35%) for VL. For 76 of the 89 (85.4%) positive samples, the diagnosis was made by microscopy showing the presence of amastigote forms on thin smears, while 13 of 89 (14.6%) samples (11 CLs and 2 VLs) were qPCR-positive only. The sensitivity of the microscopic examination compared to qPCR in our study was 86% (69/80) for CL and 78% (7/9) for VL. The samples with negative microscopic examinations had low parasitic loads (Cq ≥ 29). A Cq ≥ 29 was associated with a negative direct examination in 41% (11/27) of the CL samples and 50% (2/4) of the VL samples.

Identification was successful for all VLs (n = 9) and CLs from Latin America (n = 12), but failed for 7% (5/68) of the CLs from the Old World. The presence of PCR inhibitors to explain these failures was excluded, as was a lack of sufficient clinical material (at least 10^3^ human cells/sample). The failed identifications were attributed to insufficient parasitic loads in the samples. Indeed, the five failures were all among the 32 samples with a Cq > 29, and all were sampled more than 90 days after the initial skin lesions appeared.

### Clinical presentation

#### Patients with VL

Nine patients had VL; all were men, aged from 2 to 73 years. All the patients had a prolonged stay in an *L. infantum*-endemic country (Algeria, n = 5; French Mediterranean seashore, n = 2; Republic of Georgia (South Caucasia), n = 1; and Spain, n = 1). Seven of the patients had known immunodepression (AIDS, n = 3; lymphoma, n = 3; vasculitis, n = 1). The other two were a 50-year-old man lost to follow-up and a 2-year-old boy with pancytopenia, who was hospitalized for suspicion of leukemia after a travel in south-eastern Spain (Alicante region). All were treated with liposomal amphotericin B. Three patients with unsolved immunodepression had recurrences of circulating *Leishmania* DNA. Of the seven patients with known outcome, two died (one patient with AIDS and one patient with lymphoma).

The only species identified in VL was *L. infantum*. Seven sequences perfectly matched the Greek reference strain (*L. infantum* MCAN/GR/94/CRE69: GenBank access number 156 EF579913) and the Tunisian reference sequence (*L. infantum* MHOM/TN/80/IPT1: GenBank 157 access number EF579895).The remaining two sequences had a previously identified C779T synonymous SNP (His-His) [11].

#### Patients with Latin American CL (Table 1)

**Table 1:** Main clinical features of the Latin America leishmaniasis cases and results of sequencing of the cytochrome *b* fragment

	***L. guyanensis*** ** n = 7**	***L. braziliensis*** ** n = 2**	***L. mexicana*** ** n = 2**	***L. panamensis*** ** n = 1**
Visited country				
Bolivia	–	1	–	–
Costa Rica	–	–	–	1
French Guiana	5	–	–	–
Mexico	–	–	2	–
Peru	2	1	–	–
Length of stay				
< 30 days	6	1	1	1
30–90 days	–	1	–	–
> 90 days	1	–	1	–
Occurrence of skin lesions				
< 30 days after return	5	2	1	–
30–100 days after return	2	–	1	1
Delay for seeking medical assistance				
≤ 90 days	3	2		1
> 90 days	4		2	
Exudative lesions	4	2	0	1
Treatment^a^				
Pentamidine	4	–	–	1
Intralesional meglumine antimoniate	–	1^b^	1	–
Liposomal amphotericin B	2	1^b^	–	–
Itraconazole	1^c^	–	–	–
Cytochrome b sequencing				
Reference sequence GeneBank access number	AB095969.1	LC472861.1	AB095963.1	MK570510
SNP	Absence	Absence	C53T (Met- > Thr) and A584G (Gln- > Gln)	absence

^a^Two patients (1 *L. braziliensis* and 1 *L. mexicana*) were included in therapeutic trials in another hospital

^b^Same patient treated with an association intralesional meglumine antimoniate and liposomal amphotericin B

^c^Patient treated with itraconazole because of previous treatment with pentamidine in South America

Twelve patients (9 men, 3 women; mean age 45 ± 14 years) were diagnosed with CL after trips in Latin America. The cutaneous lesions were all on exposed skin and consisted of seven exudative ulcers and five crusty skin lesions without mucosal involvement. All were positive on microscopy examination. The identified species were 7 *Leishmania guyanensis* (n = 7), 2 *Leishmania braziliensis* (n = 2), *Leishmania mexicana* (n = 1), and *Leishmania panamensis* (n = 1). The mean delay between lesion appearance and medical advice was 79 ± 38 days, with no differences between the species involved. When present, the DNA sequences showed synonymous mutations (Table 1). Treatment protocols were in accordance with consensual recommendations [6]. No patients developed mucosal lesions.

#### Patients with Mediterranean CL

Cutaneous leishmaniasis from the Mediterranean basin was diagnosed in 68 patients (median age: 46 years, range 1–83; 43 males, 25 females). In 88% (60/68) of the cases, the patients were of African origin and travelled regularly to their or their relatives’ native countries during summer vacation. The patients aged < 30-years-old (n = 26) were mainly born in non-endemic areas (69%; 18/26), while those ≥ 30-years-old (n = 42) were primarily (88%; 37/42) long-term residents in France who were born in endemic areas. The median length of stay in the endemic region was 84 days (range 20–365 days). When the information was available, the cutaneous lesions appeared either during their stay (33%; 18/54) or within a month of their return from the endemic area (37%; 20/54). However, the median day between appearance of the lesions and diagnosis was 90 [60–127]. Thus, despite the usual onset of lesions within a month of returning, most of the patients delayed seeking medical advice for more than two months after the return from endemic area.

The most frequently visited regions associated with CL were North Africa [Tunisia, n = 25 (more specifically the Tataouine region n = 8); Morocco, n = 8; Algeria, n = 6] and West Sub-Saharan Africa (Senegal, n = 11; Mauritania, n = 7; Mali, n = 3). We also had cases imported from the Middle East (Israel n = 3, with 2 children in the same family; Egypt, n = 1), and Southern Europe (Spain n = 3; Italy n = 1).

Three species were identified in 93% (63/68) of the patients. The sequencing failed in 5 patients (2 from Algeria and 1 each from Mauritania, Spain, and Italy) and corresponded with those showing a low parasitic burden in skin lesions that had lasted more than 90 days. *Leishmania major* was by far the most frequent (48/63; 76%) species identified, followed by *L. tropica/killicki* (9/63; 14%), and *L. infantum* (6/63; 10%).

The median length of stay in the endemic regions was 60 days (range 20–365 days), with no significant differences between the three species (Table 2). Because the journeys to endemic areas mainly took place during summer vacation, the monthly distribution of the CL diagnoses in our center was between October and February (n = 47), with a peak in January (n = 16).

**Table 2:** Clinical and epidemiologic characteristics of the 63 (out of 68) Mediterranean CL patients for who the *Leishmania* species was identified

	*L. major* n = 48 (76%)	*L. tropica/killicki *n = 9 (14%)	*L. infantum* n = 6 (10%)
Median age years [IQ25–IQ75]	39 [16.3–56.5]	42 [12–57]	56 [22–56]
Sex ratio (M/F)	1.66	1.25	2
Median length of stay in endemic area days [IQ25-IQ75]	60 [30–90]	60 [48–60]	45 [30–75]
Occurrence of skin lesions (n/data available)			
During the stay	12/38 (32%)	2/8 (25%)	2/4 (50%)
≤ 30 days after the return	16/38 (42%)	2/8 (25%)	1/4 (25%)
> 1 to ≤ 3 months	8/38 (21%)	3/8 (37%)	0/4 (0%)
> 3 months	2/38 (5%)	1/8 (13%)	1/4 (25%)
Median days before seeking medical advice [IQ25-IQ75]	90 [60–103]	90 [90–12]	180 [128–255]
Number of lesions (n/data available)			
Unique	18/47 (38%)	5/9 (56%)	3/6 (50%)
Multiple	29/47 (62%)	4/9 (44%)	3/6 (50%)
Localization of lesions (n/data available)			
Limb (at least one lesion)	37/46 (80%)	3/9 (33%)	3/6 (50%)
Face (at least one lesion)	9/46 (20%)	6/9 (67%)	3/6 (50%)
Crusted ulcero-necrotic lesions	44/48 (92%)	6/9 (67%)	1/6 (17%)
Country of contamination			
North- Africa (n = 37)	24/37 (65%)	9/37 (24%)	4/37 (11%)
Algeria (n = 4)	2/4	–	2/4
Morocco (n = 8)	4/8	2/8	2/8
Tunisia (n = 25)	18/25	7/25	–
Sub-Saharan Africa (n = 20)	20/20 (100%)	–	–
Mali (n = 3)	3/3	–	–
Mauritania (n = 6)	6/6	–	–
Senegal (n = 11)	11/11	–	–
Middle East (n = 4)	4/4 (100%)	–	–
Egypt (n = 1)	1/1	–	–
Israel (n = 3)	3/3	–	–
Europe (n = 2)	–	–	2/2 (100%)
Spain (n = 2)	–	–	2/2

**Fig. 1:**
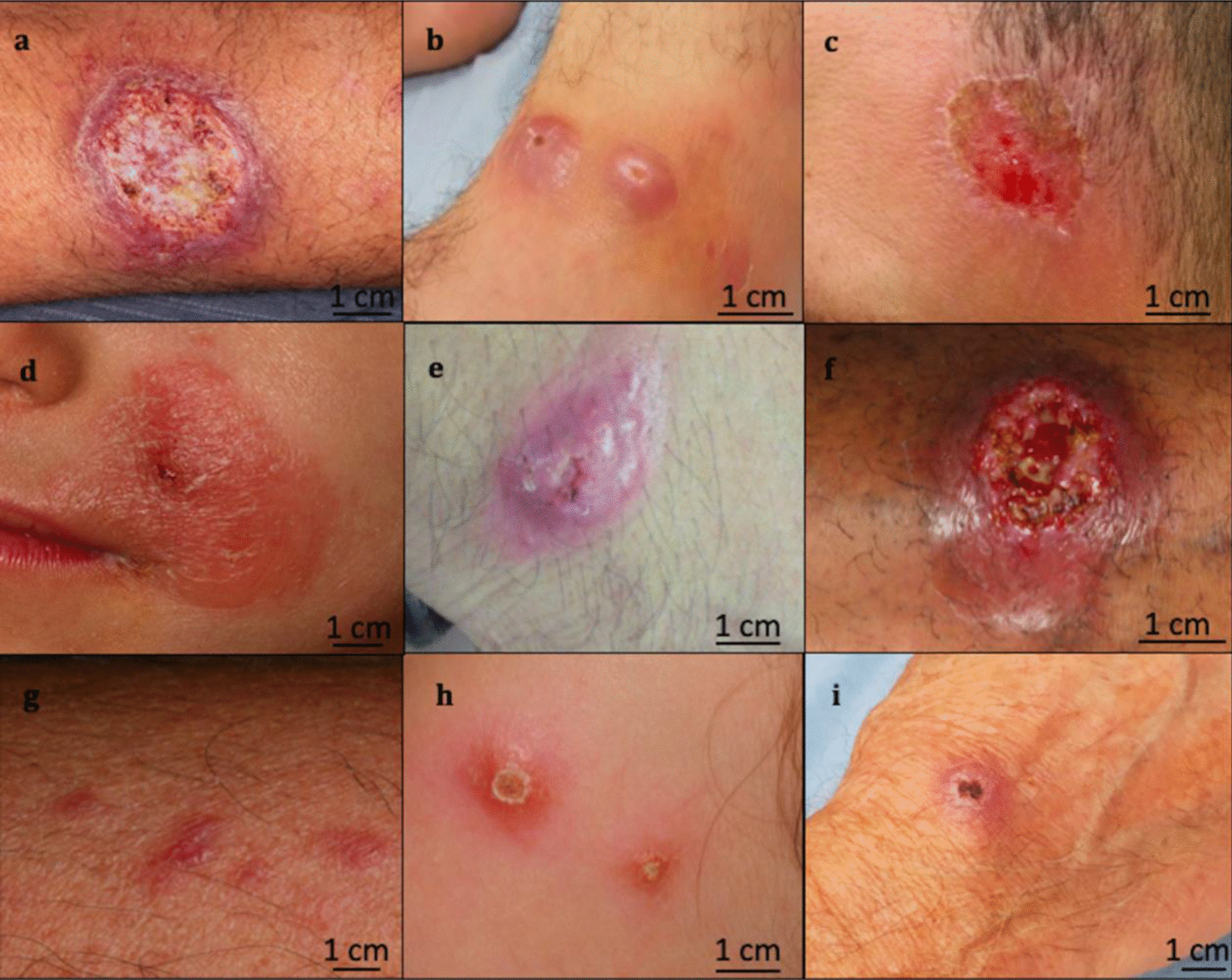
Clinical appearance of Mediterranean cutaneous leishmaniasis lesions according to the identified species showing the clinical polymorphism. *Leishmania major*: **a** typical crusty ‘pizza-like’ lesion; **b** atypical erythemato-papulous lesions; **c** atypical inflammatory lesion. *Leishmania tropica/killicki*: **d** typical face-located lesion; **e** and **f** atypical limb-located inflammatory lesions. *Leishmania infantum*: **g** and **h** typical inflammatory lesions; **i** atypical crusty lesion. Please note that the scale used in this figure is approximate

The correspondence of the morphological aspects of the CL lesions to the identified species is illustrated in Fig. 1. When more specifically comparing *L. major* and *L. tropica/killicki*, the clinical lesions from *L. major* were more often crusted and ulcero-necrotic (44/48 [97%] vs 6/9 [67%]; p = 0.036) and more often localized on the limbs than on the face (p = 0.004). Although the *L. major* lesions tended more often to be multiple (62%; 29/47) than the *L. tropica/killicki* lesions (44%; 4/9), the difference was not statistically significant, nor was the delay in seeking medical assistance (Table 2).

The sequenced portion of the *cytb* gene of CLs caused by *L. infantum* was identical to the sequences of the VL cases. Sequences identified as *L. tropica* (n = 3, imported from Tunisia and Morocco) matched with two reference strains [*L. tropica* MHOM/SU/58/Strain OD (GenBank access number AB095960) and *L. tropica* MHOM/SU/74/K27 (World Health Organization reference strain of *L. tropica*, GenBank access number HQ908270.1 and KY360314.1)] with a query cover exceeding 99%. The 6 sequences identified as *L. killicki*, all from Tunisia, matched the *L. killicki* reference strain MHOM/TN/86/LEM163 (GenBank access number AB434676) with a query cover exceeding 99%. The 48 *L. major* sequences had a query cover exceeding 99%. with the *L. major* reference strain (GenBank access number AB095961). Among them, the 20 sequences from the patients who travelled to Sub-Saharan Africa had an A108G synonymous mutation (Val-Val) compared with the *L. major* strain (GenBank access number AB095961) reference sequence, that was not observed in the other patients. In addition, a C624T synonymous mutation (Arg-Arg) was observed in all (24/24) the *L. major* strains from patients who travelled in North Africa (Algeria, Morocco, and Tunisia), which was not present in the sequences from those who returned from Sub-Saharan Africa (20) or in three of the four patients from the Middle East. Among these later, one Israeli patient had professional travels in a lot of countries of the Mediterranean basin and a precise location for the infection could not be assessed.

## Discussion

We report on a comprehensive collection of 89 leishmaniasis cases seen over more than 7 consecutive years in a university hospital located in Paris, France. These cases consisted of 77 Old World and 12 New World *Leishmania* cases. The cohort primarily included CLs from North Africa (39 cases) or West Sub-Saharan Africa (21 cases).

Diagnostic qPCR targeting the 18S rRNA gene [11] was more sensitive than microscopy for routine diagnosis, as has already been shown using other PCR gene targets and methods for CL [14] and New World *Leishmania* spp. [15]. Besides the closed tube format to avoid contamination with amplicons and thus false positive results, the main interest in qPCR is to exclude false negative results by accurately quantifying the parasitic load and determining if PCR inhibitors are present [16]. We confirmed that a CL diagnosis could be corrected when microscopy was negative, even when the lesions appeared old and were healing either spontaneously or due to previous treatment. For VL diagnoses, a more sensitive tool than microscopy is crucial for proper diagnosis and follow up.

A different target was chosen for the identification step than for the diagnostic step, namely the *cytb* gene, as previously proposed for *Leishmania* sp. identification [17]. This precaution was designed to limit false positives caused by routine laboratory contamination. Indeed, opening amplicon-containing tubes for secondary analyses such as sequencing leads to a risk of contamination from the laboratory environment. Moreover, in contrast with non-sequence-based methods such as ITS-RFLP (internal transcribed spacer region-restriction fragment length polymorphisms), sequencing identifies SNPs that can be useful for comparison with databases [18]. This target also allows for the correct identification of *L. killicki* within the heterogeneous *L. tropica* complex [19]. However, the number of copies of the *cytb* gene (~ 50 [17]) is less than that of the 18S rRNA gene (~ 50–200 [20]), explaining why species identification failed for five samples with low parasite loads.

For VL occurring in non-endemic areas, the issue is to increase the suspicion index and confirm the diagnosis using qPCR [21]. Indeed, the clinical presentation is not specific and can be seen in different situations of immunosuppression such as HIV infection [22], anti-TNF treatment [23, 24], after solid organ transplantation [25], or after hematopoietic stem cell transplantation [26]. Excepted for HIV co-infection in endemic areas [22], the prevalence of VL is often very low [23, 24]. The accepted physiopathology is the reactivation of a persistent parasite from a previous primo-infection subsequent to an acquired immunodeficiency [27]. Thus, the disappearance of circulating DNA after treatment does not mean the parasite has been cleared from the organism, hence the possible reoccurrence of the disease [22]. Conversely, detection of leishmania DNA is not synonymous of an ongoing clinical active disease [28]. Visceral leishmaniasis is also a differential diagnosis of leukemia-like syndromes in infants living or traveling to endemic countries, as observed here for a pediatric case [29, 30].

For CL, the travel history easily differentiates patients returning from Latin America from those returning from Africa and the Middle East. In the former, the clinical lesions are often exudative, large, and prone to secondary bacterial infections. The main goal then is species identification, and we indeed identified different species, in accordance with the wealth of *Leishmania* spp. in this region [31, 32]. Importantly, none of the cases presented with mucosal lesions, confirming that patients seek medical advice soon enough after the appearance of such lesions. The treatments were diverse, according to patient and species (Table 1), but effective for all patients.

The largest contingent of patients with CL in our series was composed of migrants or French citizens born in France visiting relatives in North Africa or West Sub-Saharan countries. The delay between the appearance of lesions and the microbiological diagnosis was more than 3 months, which suggests these patients only consult when no spontaneous healing occurs. Consequently, the actual burden of CL is probably grossly underestimated. Three species responsible for the Old World CLs were identified: *L. infantum*, *L. tropica/killicki*, and *L. major*. Our clinical observations support previous studies reporting that lesions caused by *L. major* are more often multiple and located on limbs, whereas lesions due to *L. tropica* are usually single and face-localized [33]. These differences can be explained by the different vector behaviors [33]. Clinically, the *L. major* lesions were more exudative and wet than the *L. tropica/killicki* lesions, while the *L. infantum* lesions were more nodular infiltrative. Despite these clinical differences, however, there were numerous overlaps, and it is not possible to exclude a given species based on its clinical aspect. Moreover, the distribution areas of the species also frequently overlap [3]. This highlights the importance of molecular identification for both epidemiological purposes and to avoid misdiagnosing *L. infantum* CL, which can lead to secondary VL [34], or alternatively, to reinsure patients in cases of *L. major* lesions, which do not disseminate in the case of HIV infection [11].

The age distribution of the Old World CL cases showed a double peak, corresponding to children and adults, including the elderly. While the occurrence of CL is not surprising in naïve children born in a non-endemic area, the occurrence of CL in older people suggests either they were naïve because (i) the parasite was not contracted during childhood, (ii) the patients lost their immune status after long periods spent in France, or (iii) that previous exposure to the parasite is not in any way protective. Mandall et al. suggest that despite the induction of a protective immune response, secondary *L. major* infections can effectively establish themselves in a previously infected host, supporting the non-protective hypothesis [35]. Moreover, Bousslimi et al*.* have reported cases of CL not just in Tunisian children, but also in Tunisian adults (57.1%), which also supports the non-protective scenario [33]. In this series, one can underline the high number of CL cases after travel to south-eastern Tunisia (governorate of Tataouine), where both *L. major* and *L. tropica/killicki* are endemic [33]. This continual transmission of CL relates to environmental changes, which impact mammal reservoirs and sand fly populations, as well as demographic and human behavioral factors [36], factors specifically present in Tataouine [3].

Data on the molecular identification of *Leishmania* species from West Sub-Saharan Africa are scanty although numerous outbreaks have been reported, mainly based on serology surveys or clinical diagnoses [37]. In a recent study, 8 cases of *L. major* infections were reported from Mali using an end-point PCR assay [38]. Here, we added 20 confirmed *L. major* infections from West Sub-Saharan Africa. In accordance with the absence of *L. tropica* and *L. infantum* CL in this region, only *L. major* was reported [37]. Interestingly, we observed two synonymous mutations in the *cytb* gene (A108G and C624T) that distinguished the *L. major* cases from West Sub-Saharan Africa from those from North Africa and the Middle East. Using an isoenzyme analysis, a particular zymodeme (MON26) reported in Mauritania, Senegal, and Mali has previously been shown to differ from the common zymodeme (MON 25) from North Africa [39]. If our results are confirmed, this observation could serve as an epidemiological marker that is easily available using PCR.

We readily acknowledge the limits of our observational study to draw any epidemiological conclusions based on the frequency and intensity of transmission in the visited countries. We have no data on healthy returning travelers or those with self-limited and/or spontaneously healing lesions who did not seek medical advice. In addition, we cannot exclude that word-of-mouth information led to patients coming to consult in our hospital specifically, leading to a false impression of an increase in cases from a specific region.

## Conclusion

The risk of contracting leishmaniasis should be more widely known, specifically for patients of African origin visiting relatives who may believe themselves to be immune to these diseases. Indeed, while some lesions are limited and heal easily, others can be extensive with definite unaesthetic scars after healing. Since there are no current vaccines or prophylaxis recommendations, the only preventive measure is to reduce contact with sand flies by using personal protective measures such as avoiding outdoor activities, wearing protective clothing, and applying insect repellent when sand flies are most active. Sleeping in air-conditioned or well-screened areas may be a formal recommendation, but in low-income countries, fans or ventilators might be more affordable, and they do considerably inhibit the movement of sand flies, which are weak fliers [7].

From a laboratory point of view, our diagnostic strategy enables both a positive diagnosis and an accurate species identification [11]. For VL, a more systematic use of qPCR should be implemented in cases of unexplained fever in immunocompromised patients that have lived or travelled in endemic areas [21]. The species identification available using expensive equipment in the laboratories in high-income countries can be useful in identifying *Leishmania* species from countries where information is currently lacking [31, 40].

## Data Availability

All the data and materials are included in the manuscript.
